# Asian Citrus Psyllid Expression Profiles Suggest *Candidatus* Liberibacter Asiaticus-Mediated Alteration of Adult Nutrition and Metabolism, and of Nymphal Development and Immunity

**DOI:** 10.1371/journal.pone.0130328

**Published:** 2015-06-19

**Authors:** Meenal Vyas, Tonja W. Fisher, Ruifeng He, William Nelson, Guohua Yin, Joseph M. Cicero, Mark Willer, Ryan Kim, Robin Kramer, Greg A. May, John A. Crow, Carol A. Soderlund, David R. Gang, Judith K. Brown

**Affiliations:** 1 School of Plant Sciences, The University of Arizona, Tucson, Arizona, United States of America; 2 Institute of Biological Chemistry, Washington State University, Pullman, Washington, United States of America; 3 BIO5, The University of Arizona, Tucson, Arizona, United States of America; 4 National Center for Genome Resources, Santa Fe, New Mexico, United States of America; The University of Western Australia, AUSTRALIA

## Abstract

The Asian citrus psyllid (ACP) *Diaphorina citri* Kuwayama (Hemiptera: Psyllidae) is the insect vector of the fastidious bacterium *Candidatus* Liberibacter asiaticus (CLas), the causal agent of citrus greening disease, or Huanglongbing (HLB). The widespread invasiveness of the psyllid vector and HLB in citrus trees worldwide has underscored the need for non-traditional approaches to manage the disease. One tenable solution is through the deployment of RNA interference technology to silence protein-protein interactions essential for ACP-mediated CLas invasion and transmission. To identify psyllid interactor-bacterial effector combinations associated with psyllid-CLas interactions, cDNA libraries were constructed from CLas-infected and CLas-free ACP adults and nymphs, and analyzed for differential expression. Library assemblies comprised 24,039,255 reads and yielded 45,976 consensus contigs. They were annotated (UniProt), classified using Gene Ontology, and subjected to *in silico* expression analyses using the Transcriptome Computational Workbench (TCW) (http://www.sohomoptera.org/ACPPoP/). Functional-biological pathway interpretations were carried out using the Kyoto Encyclopedia of Genes and Genomes databases. Differentially expressed contigs in adults and/or nymphs represented genes and/or metabolic/pathogenesis pathways involved in adhesion, biofilm formation, development-related, immunity, nutrition, stress, and virulence. Notably, contigs involved in gene silencing and transposon-related responses were documented in a psyllid for the first time. This is the first comparative transcriptomic analysis of ACP adults and nymphs infected and uninfected with CLas. The results provide key initial insights into host-parasite interactions involving CLas effectors that contribute to invasion-virulence, and to host nutritional exploitation and immune-related responses that appear to be essential for successful ACP-mediated circulative, propagative CLas transmission.

## Introduction

The Asian citrus psyllid (ACP), *Diaphorina citri* Kuwayama (Hemiptera: Psyllidae) is the insect vector and host of *Candidatus* Liberibacter asiaticus (CLas), the causal agent of citrus greening disease, also known as Huanglongbing (HLB) [1–3]. CLas is a fastidious, phloem-limited bacterium classified into the α subdivision of the *Proteobacteria* [4]. The ACP-CLas complex is endemic to Asia, and exotic to all citrus growing regions of Africa, the Caribbean Basin, Central, North, and South America, the Mediterranean region, and the Arabian Peninsula [5–7] (http://www.aphis.usda.gov). In the U.S. CLas was first detected in citrus trees in Florida during 2004–05, following the introduction of ACP there ten years before [3]. Since then, CLas has been confirmed infecting citrus trees in California, Florida, Georgia, Louisiana, South Carolina, and Texas (http://www.hungrypests.com/faqs/citrus-greening.php).

The biology of *Ca*. Liberibacter interactions with its psyllid vector are not well studied at the functional genomics level. CLas and several other Liberibacter species infect both their psyllid vector and a suite of plant hosts, typically in a plant host-vector specific manner [8–13]. Based on the relatively small genome size of CLas 1.23-Mb [14], the absence of plant-colonizing extracellular degradative enzymes, and the predicted, limited ability for aerobic respiration, the bacterium is predicted to utilize key host plant and probably psyllid metabolites, and has adopted an intracellular lifestyle with the host plant like its close relatives in the Rhizobiales [15], and has evolved a host-parasite relationship akin to certain other pathogens that engage in multi-partite interactions to enable dual host exploitation [16].

The transmission of CLas by ACP adults reared from the egg to adult stage on CLas-infected citrus host plants occurs at a frequency of ~40% [17]. However, CLas transmission has been demonstrated to be efficient, only when the bacterium is ingested by the nymphal stages of ACP [18]. Whether a particular nymphal stage or stages must ingest CLas to facilitate adult-mediated transmission has not been determined. Although CLas-free adults that have been given an acquisition-access period (AAP) on CLas-infected plants harbor the bacterium (e.g. based on molecular detection), transmission of CLas to the plant host has not been demonstrated [17, 18]. In addition, the detection of CLas by polymerase chain reaction (PCR) in the offspring of CLas-infected ACP reared on infected citrus, at a frequency of 2–6% [17], indicates that transovarial (vertical) transmission is not the primary mode by which CLas sustains its relationship with the psyllid or plant host, but rather serves as a fail-safe mechanism to ensure its survival in a small fraction of the ACP population when the ACP-reproductive hosts are unavailable. A similarly low frequency of sexual transmission was also reported to occur between males and females [19] and could likewise serve as a survival strategy during reproductive diapause [20].

Comparisons of the CLas genome with intracellular parasites of other insects and mammals [15] indicate it encodes no genes for purine and pyrimidine metabolism, and has a minimal number of DNA excision repair genes, suggesting that CLas depends on the psyllid host for essential metabolic functions and nutritional requirements uniquely, in relation to those provided during the plant host portion of the cycle. This is consistent with other well-studied symbioses involving insect-microbe complexes that share nutritional resources and proteins essential for survival by partitioning them between both partners [21].

Virulence factors of vertically-transmitted, propagative bacteria are essential for invasion and establishment in the host, and comprise carbohydrates, lipids, or proteins that function either as toxins or effectors. Gram negative bacterial effectors are delivered directly or indirectly to the host cell, typically as toxins or through secretion systems to exploit host proteins during invasion, colonization, and systemic spread [22–24]. Certain plant pathogen-insect vectors transmit plant viruses and fastidious bacteria that are circulative and propagative in the insect. In these instances pathogen-encoded effector proteins are essential for infection and circulation in the insect vector, and utilize a mode of transmission referred to as circulative and propagative [25], making them pathogenic to both insect and plant hosts. Only somewhat recently, CLas as well as certain other psyllid-associated Liberibacter species have been shown to use a circulative, propagative mode of transmission. In these systems the bacterium associates with the psyllid gut, filter chamber, hemolymph, salivary glands, muscles, and fat bodies [8–11].

**Fig 1:**
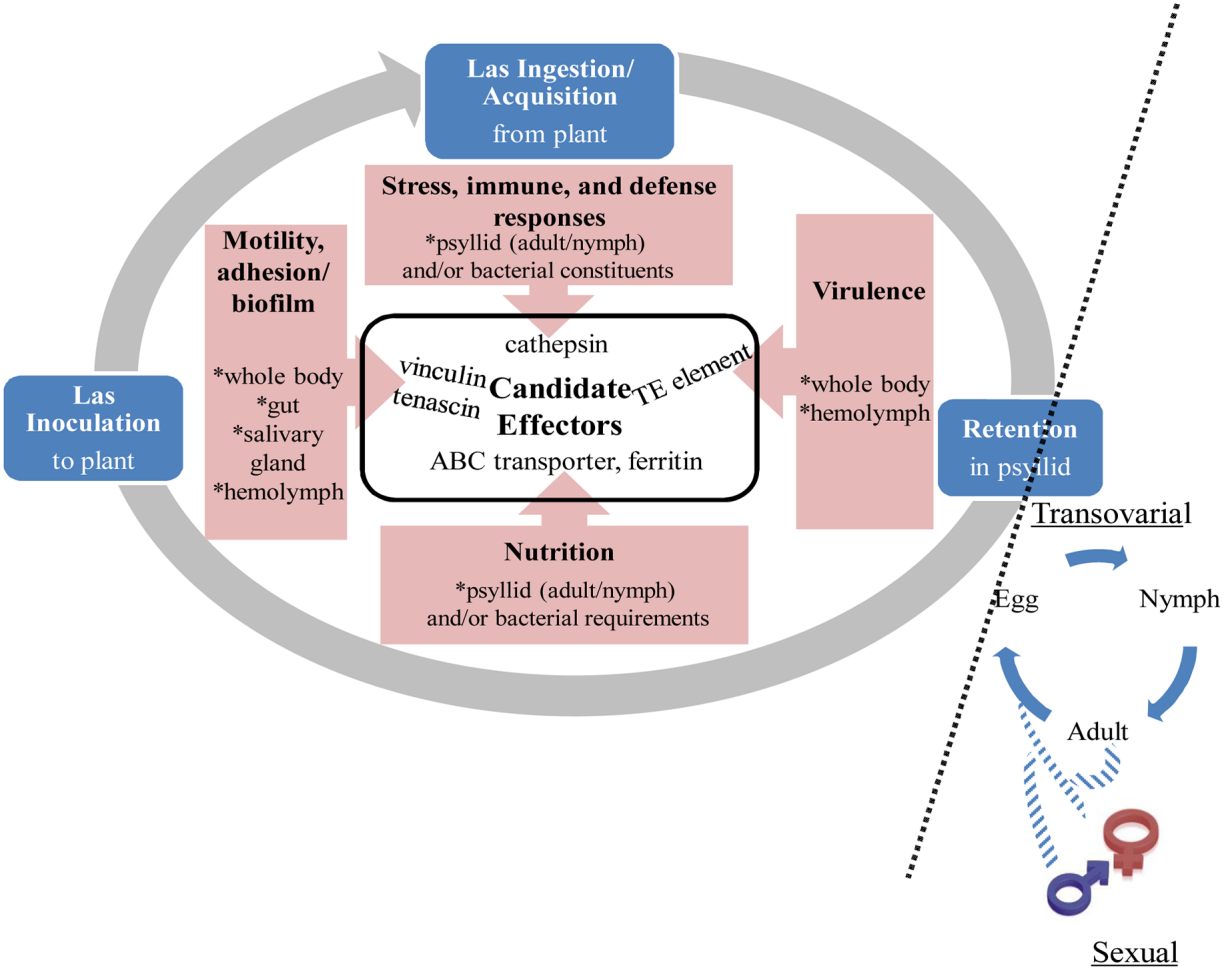
Proposed model of the circulative, propagative transmission pathway and selected hypothetical effectors involved in ACP responses to CLas infection, multiplication, circulation, and acquisition of ACP based on the comparative expression profiles.

Genetic manipulation and other strategies for managing vector-borne pathogens [26–28], have been deployed to interfere with vector-mediated transmission of viruses. In one example virus-vector competency of mosquitos for Dengue virus has been reduced substantially by infecting the vector with Wolbachia (wMel), which blocks virus replication, and makes the mosquito refractory to transmission to the human host [27]. This and other recent successes have stimulated an interest in the use of non-genetic approaches such as dsRNA technology to mediate gene knock down in RNAi-competent insects, among which are ACP and the potato psyllid [29–30]. The circulative-propagative CLas transmission cycle (Fig 1) involves the ingestion of CLas by ACP from plant sap, which passes into the food canal and then the alimentary canal (gut) where CLas resides, multiplies, and forms extensive biofilms [31]. CLas exits the gut to enter the hemolymph where it becomes motile [32–33] and circulates in the blood to the oral region and enters the salivary glands, the presumed organ of transmission specificity. The dsRNA targeting of ACP transcripts that interact with CLas effectors during the invasion-adhesion-biofilm formation, multiplication, circulation, and acquisition phases, hinges on the ability to identify those that respond specifically to CLas in the circulative, propagative pathway in its psyllid vector. Interactors responsible for transovarial and sexual transmission might also play an important role in fitness of the pathogen because it guarantees transmission by alternate means. Therefore it would be ideal to look at the transmission cycle as a whole to dissect out the effectors involved at different stages (Fig 1). So far, only stage and tissue specific transcripts have been documented from CLas-free ACP by Hunter et al. [34] and Reese et al. [35].

The objective of this study was to identify differentially expressed candidate transcripts in cDNA libraries constructed from ACP adults and nymphs infected or uninfected with CLas ACP, whose expression, if reduced or abated altogether, might impede CLas-psyllid host interactions essential for viable circulative, propagative transmission of CLas, thereby preventing transmission to the plant host.

## Materials and Methods

### Psyllid colonies

The ACP CLas-infected and—uninfected colonies were reared in laboratory cultures maintained on a CLas host (*Citrus* spp.) (CLas-infected) or a CLas- immune rutaceous plant species (CLas-free). Cultures were reared continuously and serially transferred periodically to the same host species at the University of Florida Citrus Research and Education Center (courtesy, Dr. K.S. Pelz-Stelinski, Lake Alfred, FL) or at the Southwest Florida Research and Education Center (courtesy, Dr. P.A. Stansly, Immokalee, FL). Adults and nymphs (2–5 instar) were collected so that the most complete whole transcriptome data set could be generated over a complete range of adult and nymph life stages representing complete cohorts, minus the first instar, which was too small to obtain near-equal body weight in comparison to the other nymphal instars. Live psyllids were collected from colonies and processed by crushing the bodies lightly in RNA-free tubes using a micro-pestle, followed by the addition of Trizol. Samples were shipped on dry ice to Washington State University where they were stored at -80°C until use.

### Total RNA isolation and quality control

Total RNA was extracted from 100 CLas-infected (WbL) and—uninfected (Wb) ACP adults and from 225 CLas-infected (NyL) and—uninfected (Ny) ACP nymphs, respectively. Samples were ground in liquid nitrogen with a micro-pestle followed by resuspension in 1 ml Trizol (Invitrogen, Carlsbad, CA) each. For RNA extraction, 0.3 ml chloroform were added to 1 ml Trizol homogenate, followed by vigorous sample shaking for 30s, which was then allowed to sit for 3 min at room temperature. The samples were centrifuged at 12,000 ×g for 15 min at 4°C to separate the organic from the aqueous phase. The aqueous phase (200–250 μl) was transferred to a sterile, RNase-free tube, and an equal volume of 100% RNA-free ethanol was added and mixed. The RNA was purified using the RNeasy Mini Kit (Qiagen, Valencia, CA), according to the manufacturer’s instructions. The quality of the psyllid RNA was analyzed using a NanoDrop 2000 Spectrophotometer (Thermo Scientific, Wilmington, DE) and the optical density readings were used to calculate the A260/A280 ratio (~2.0). The RNA was quantified on an Agilent Bioanalyzer 2100 RNA Nanochip (Agilent Technologies Inc., Santa Clara, CA) to ensure sufficient RNA (minimum, 2 μg) was available for cDNA synthesis and Illumina DNA sequencing for library construction.

### Library construction and Illumina sequencing

Poly(A) RNA was isolated from 2 μg of total RNA purified from each sample using magnetic oligo (dT) beads. The mRNAs were fragmented into short sequences by treatment with a zinc-containing solution at 94°C for 5 min according to the manufacturer’s instructions. The cleaved poly(A) RNA was transcribed to synthesize the first strand cDNA using random primers, followed by second-strand cDNA synthesis, end-repair and phosphorylation, and the addition of an ‘A’ base to the 3ʹ blunt end of the phosphorylated DNA fragments. The cDNA was ligated to Illumina Paired-end adapters using Illumina’s kit following manufacturer’s recommendations. The adapter-ligated products were purified on a gel to select a template size range of 250 bp ± 25 bp for downstream enrichment. The cDNA fragments were amplified using the PCR primers, PE 1.0 and PE 2.0 (Illumina), which are designed to anneal to the ends of the adapters, using the PCR program of 30 s at 98°C followed by 15 cycles of 10 s at 98°C, 30 s at 60°C, 15 s at 72°C and a final elongation step of 5 min at 72°C. The products were purified using the QIAquick PCR Purification Kit (Qiagen) to construct an Illumina paired end library. Library quality control was performed with a Bioanalyzer DNA 1000 Chip Series II (Agilent). Analysis by qPCR was employed to quantify the libraries before generating the clusters. The paired-end libraries were applied to a flowcell on a cBOT (Illumina) to generate clusters, and subjected to DNA sequencing using a 2×54 bp format on an Illumina Genome Analyzer II at **the National Center for Genome Resources** (Santa Fe, New Mexico 87505 USA). The complete dataset has been deposited to the *Short Read Archive* (SRA) at GenBank, as the Accession numbers PRJNA243541, SRX525230, SRX525218, SRX525209 and SRX525152.

### Assembly and annotation of Illumina sequences

The Illumina reads were cleaned and assembled as described by He et al. [36]. Briefly, the reads were assembled with ABySS [37], the gaps were filled using the GapCloser in SOAP [38], the scaffolds merged with Mira [39] and filtered for redundancies with Cd-hit [40], and finally, the reads were aligned *post hoc* to the final contig consensus sequences using Burrows-Wheeler algorithm (BWA) [41].

The contigs and read counts per library were analyzed using TCW software [42]. Contigs were annotated using an E-value cutoff of 1E-10 against an invertebrate, bacterial, and virus database derived from the UniProt database, and the GO terms extracted from the “.dat” files [43]. The significantly differentially expressed contigs for individual contigs and those grouped in GO categories were determined by edgeR [44] and GOSeq analysis [45], respectively.

**Fig 2:**
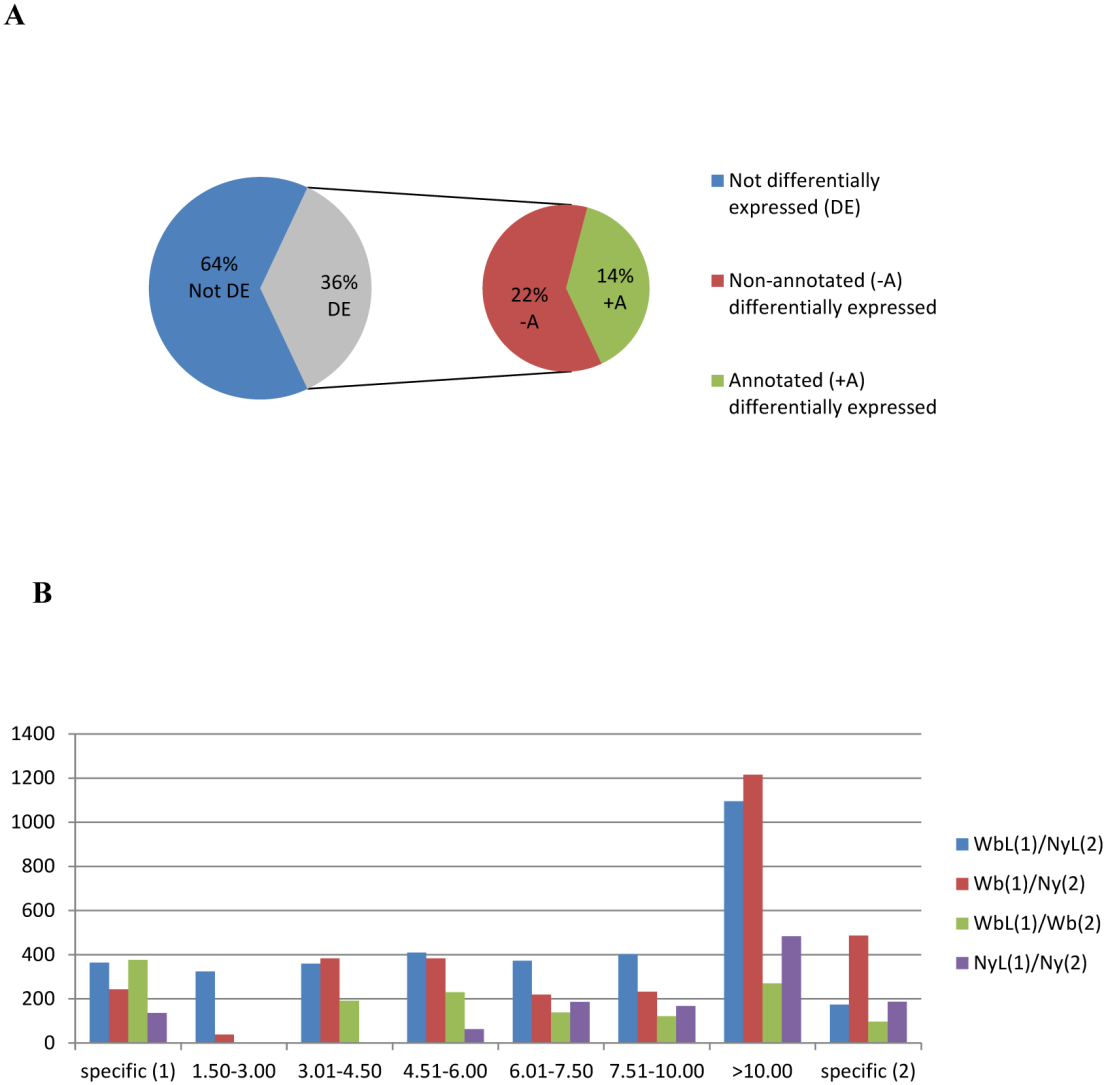
The number and distribution of differentially expressed (*p*<0.05) contigs for CLas-infected (L) and uninfected (no label) adults (Wb) and nymphs (Ny) of the Asian citrus psyllid (ACP). Pairwise comparisons were carried out for the following combinations: adults, uninfected and infected (Wb x WbL), nymphs, uninfected and infected (Ny x NyL), uninfected adult and nymphs (Wb x Ny), and infected adults and nymphs (WbL x NyL). For each comparison, the term represents the first library in each respective comparison. The number 1 refers to: Wb, Ny, Wb, and WbL, and the number 2: refers to WbL, NyL, Ny, and NyL.

**Fig 3:**
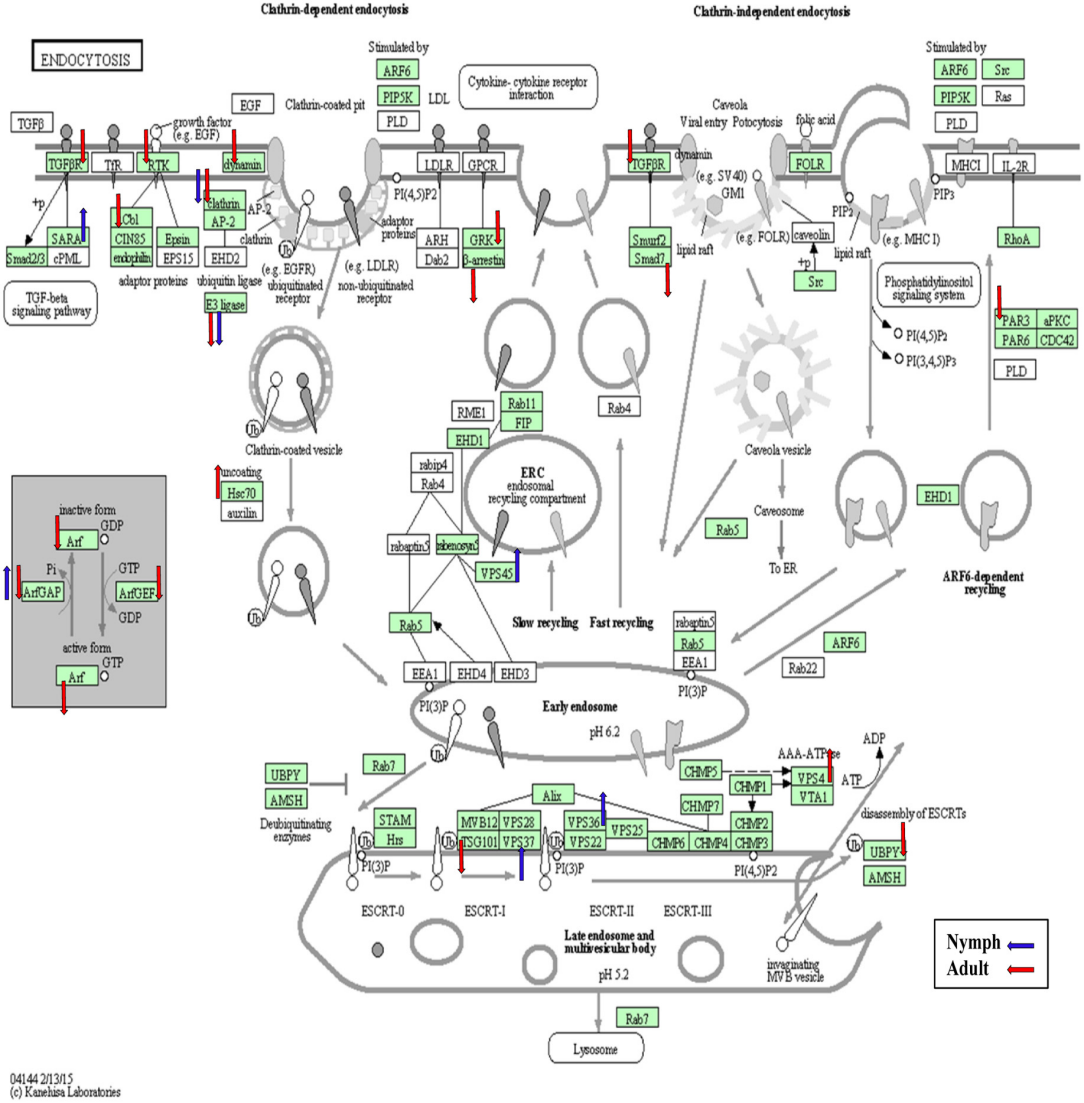
The *Endocytosis* KEGG pathway diagram showing the contigs that were differentially expressed (*p*<0.05) in nymphs (blue arrows) and adults (red arrows) in response to CLas infection. Of the assigned contigs (shaded in green), 22 were differentially expressed either in the nymphs or adults.

**Fig 4:**
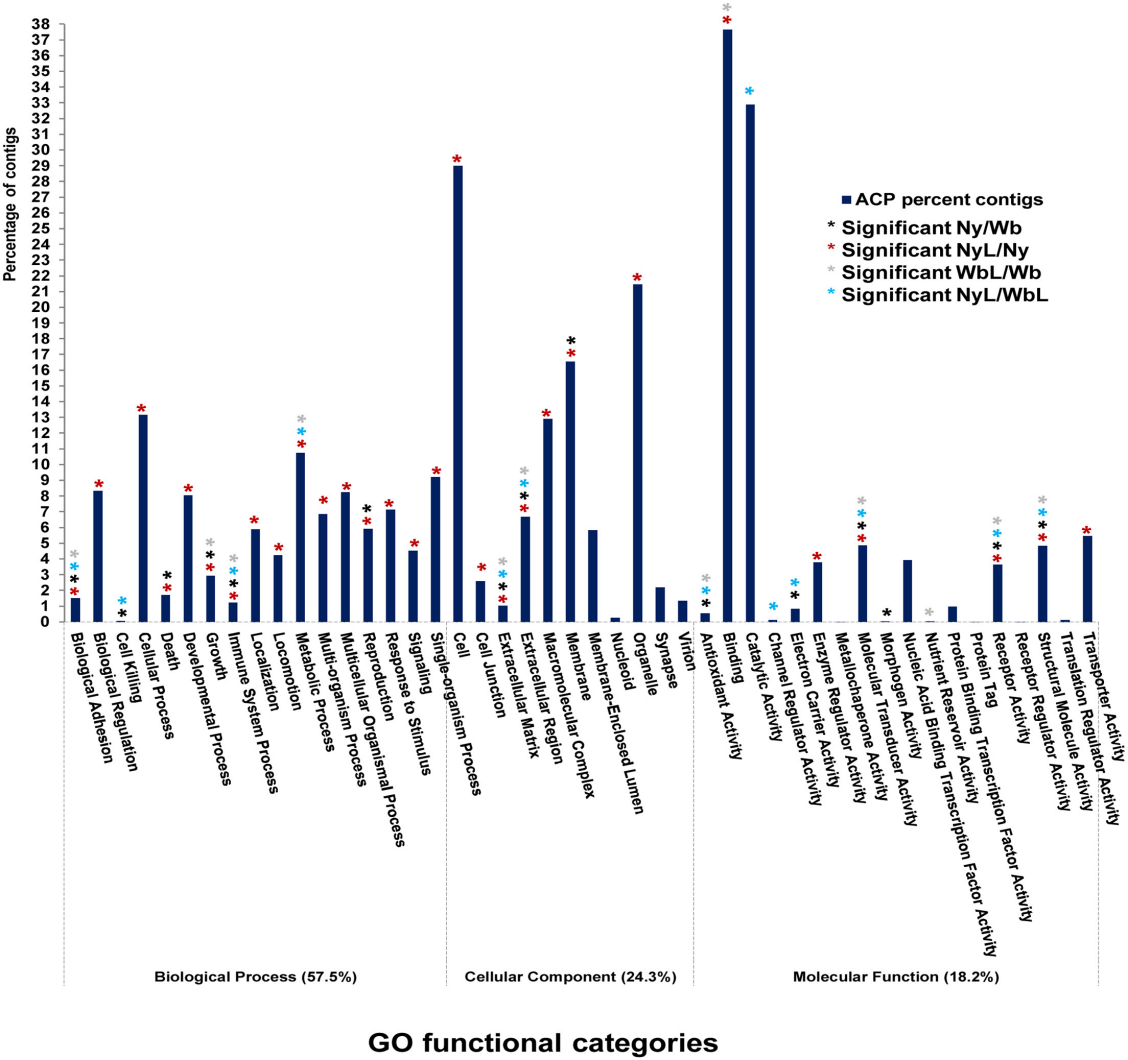
The Gene Ontology classification of annotated, differentially expressed Asian citrus psyllid (ACP) contigs, with altered expression in the presence or absence of *Ca*. Liberibacter asiaticus (CLas). The y-axis (blue bar) shows the percentage distribution of the contigs in each functional category, and the x-axis shows the 46 functional categories to which ACP contigs were assigned. Categories that contain a significant number of differentially expressed contigs are represented by an asterisk* above the blue bar, for each pairwise comparison: ACP nymphs x adults (Ny/Wb-black asterisk), infected x uninfected nymphs (NyL/Ny- blue asterisk), infected x uninfected adults (WbL/Wb—red asterisk), and infected nymphs x adults (NyL/WbL—grey asterisk). The GO classifications for contigs that showed significantly different expression levels were identified using *p*-value cut off 0.05, which was based on edgeR statistics from which selected genes have been considered as prospective candidate effectors relevant to the ACP-CLas circulative, propagative transmission pathway.

**Fig 5:**
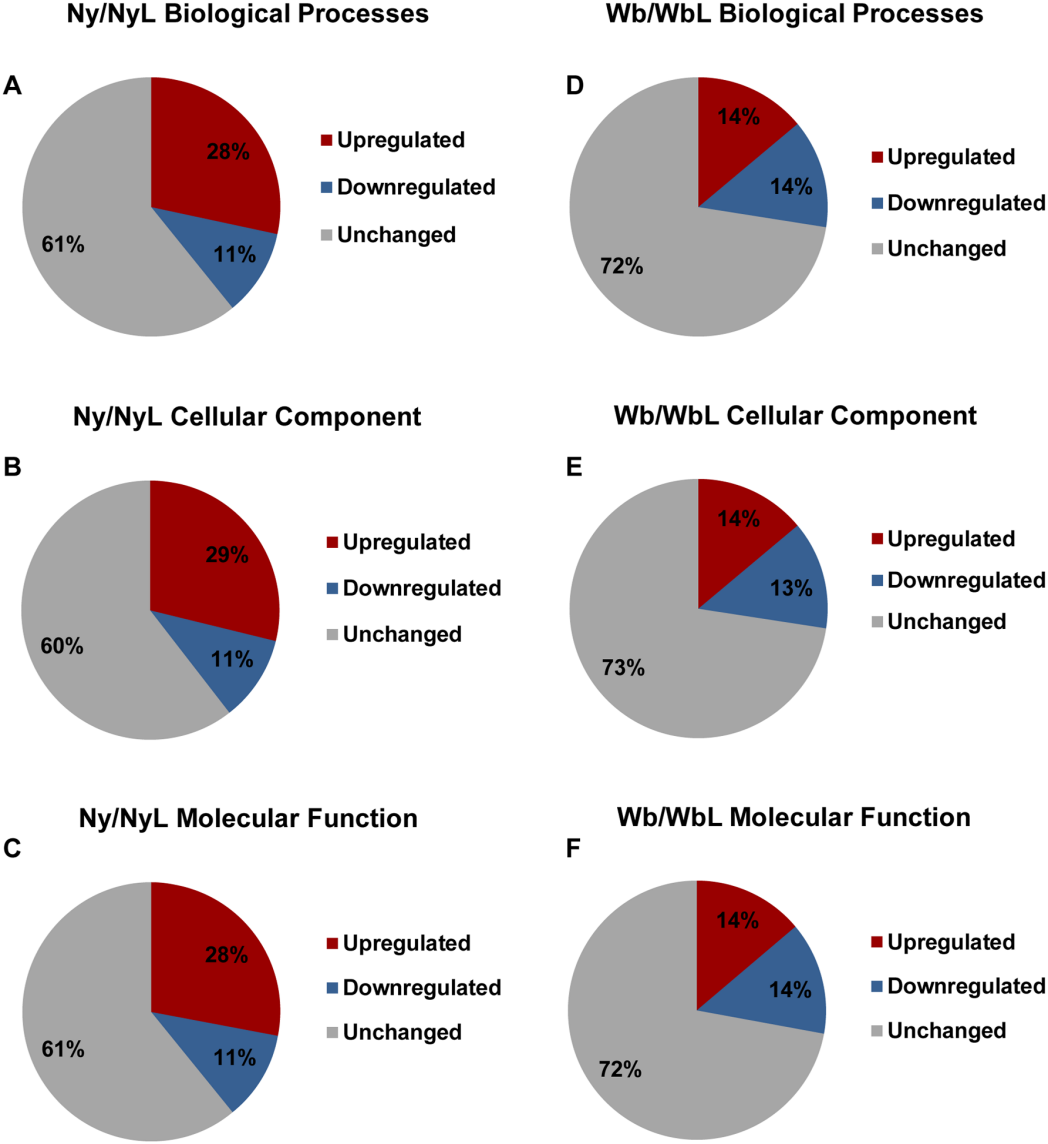
The distribution of up- or down-regulated genes among the major Gene Ontology (GO) categories. (A-C), showing the proportion of color-coded Asian citrus psyllid contigs for each group, as follows: up-regulated (red), down-regulated (blue), and unchanged (grey), in response to *Ca*. Liberibacter asiaticus (CLas) infection of nymphs (Ny), based on the Biological Process, Cellular Components and Molecular Functions GO categories. Distribution of contigs with altered expression in the GO categories, Biological Process, Cellular Components and Molecular Functions (D-F) of up-regulated (red), down-regulated (blue), and unchanged (grey) candidates, in response to CLas infection of ACP adults (Wb). Contigs classified into GO categories were identified as differentially expressed using *p*-value cut off 0.05 (edgeR statistics).

**Fig 6:**
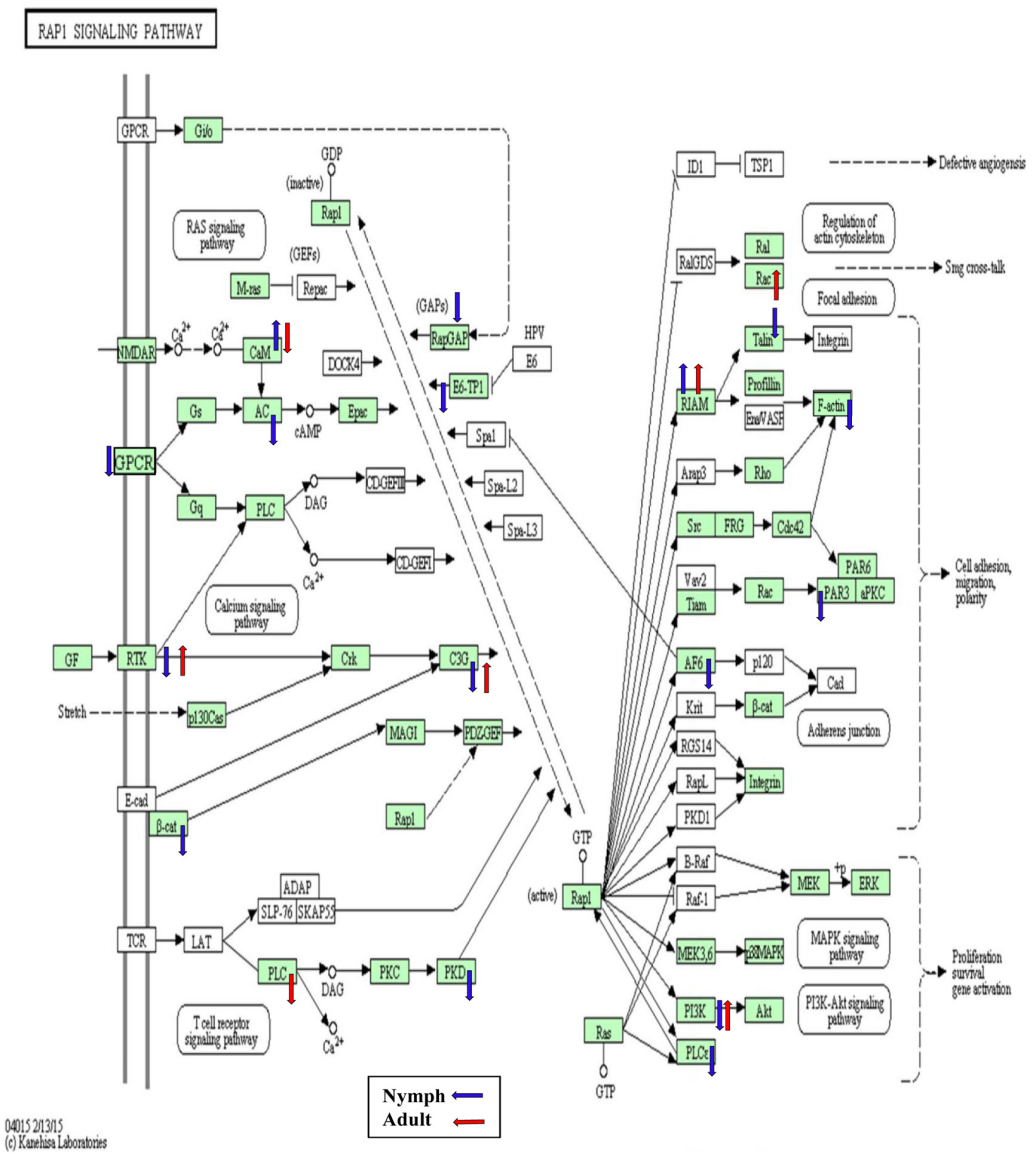
The *Rap 1 signaling* KEGG pathway diagram showing the contigs that were differentially expressed (*p*<0.05) in nymphs (blue arrows) and adults (red arrows) in response to CLas infection. Of the assigned contigs (shaded in green), 18 were differentially expressed either in the nymphs or adults.

**Fig 7:**
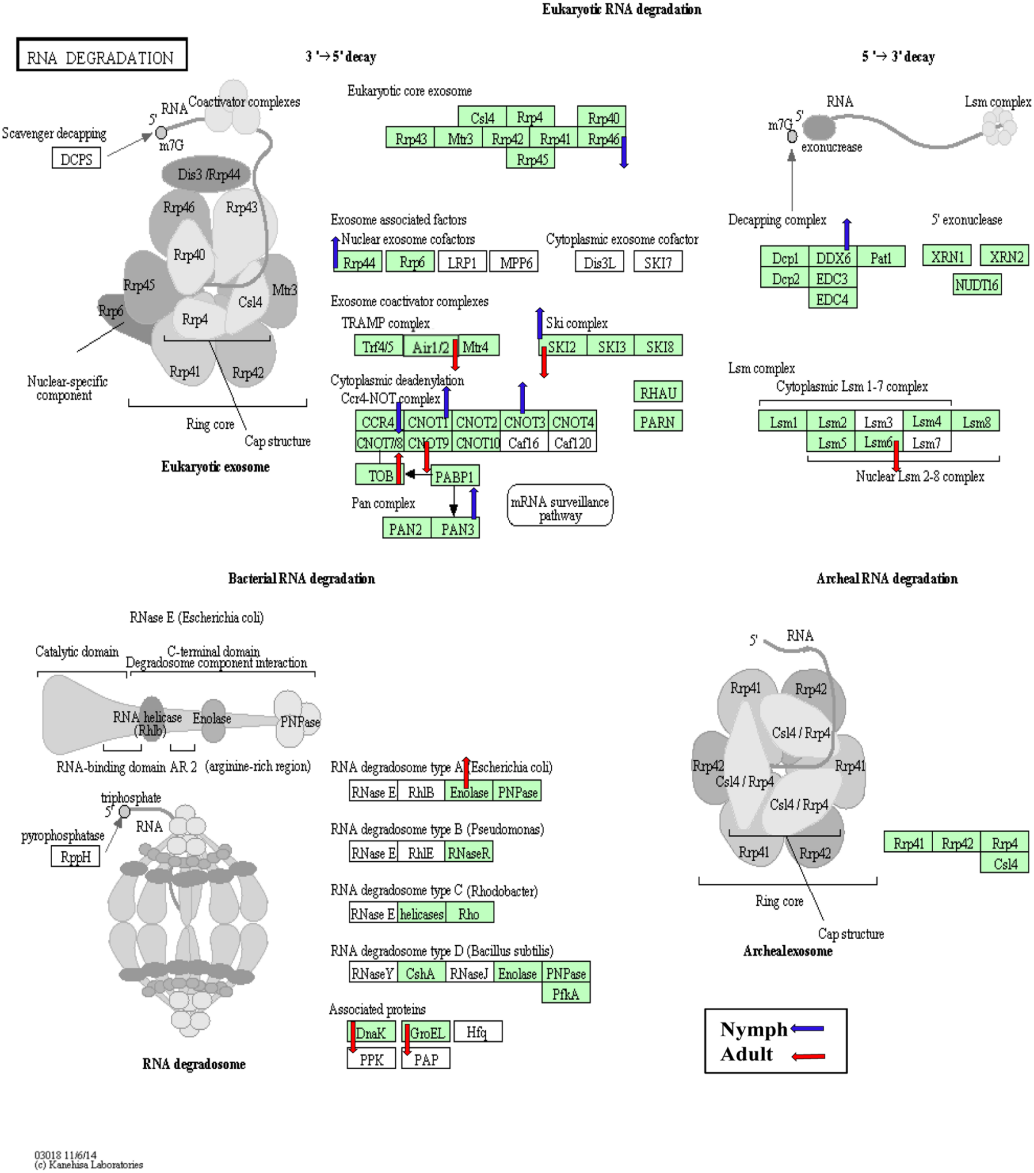
The *RNA degradation* KEGG pathway diagram showing the contigs that were differentially expressed (*p*<0.05) in nymphs (blue arrows) and adults (red arrows) in response to CLas infection. Of the assigned contigs (shaded in green), 17 were differentially expressed either in the nymphs or adults.

**Fig 8:**
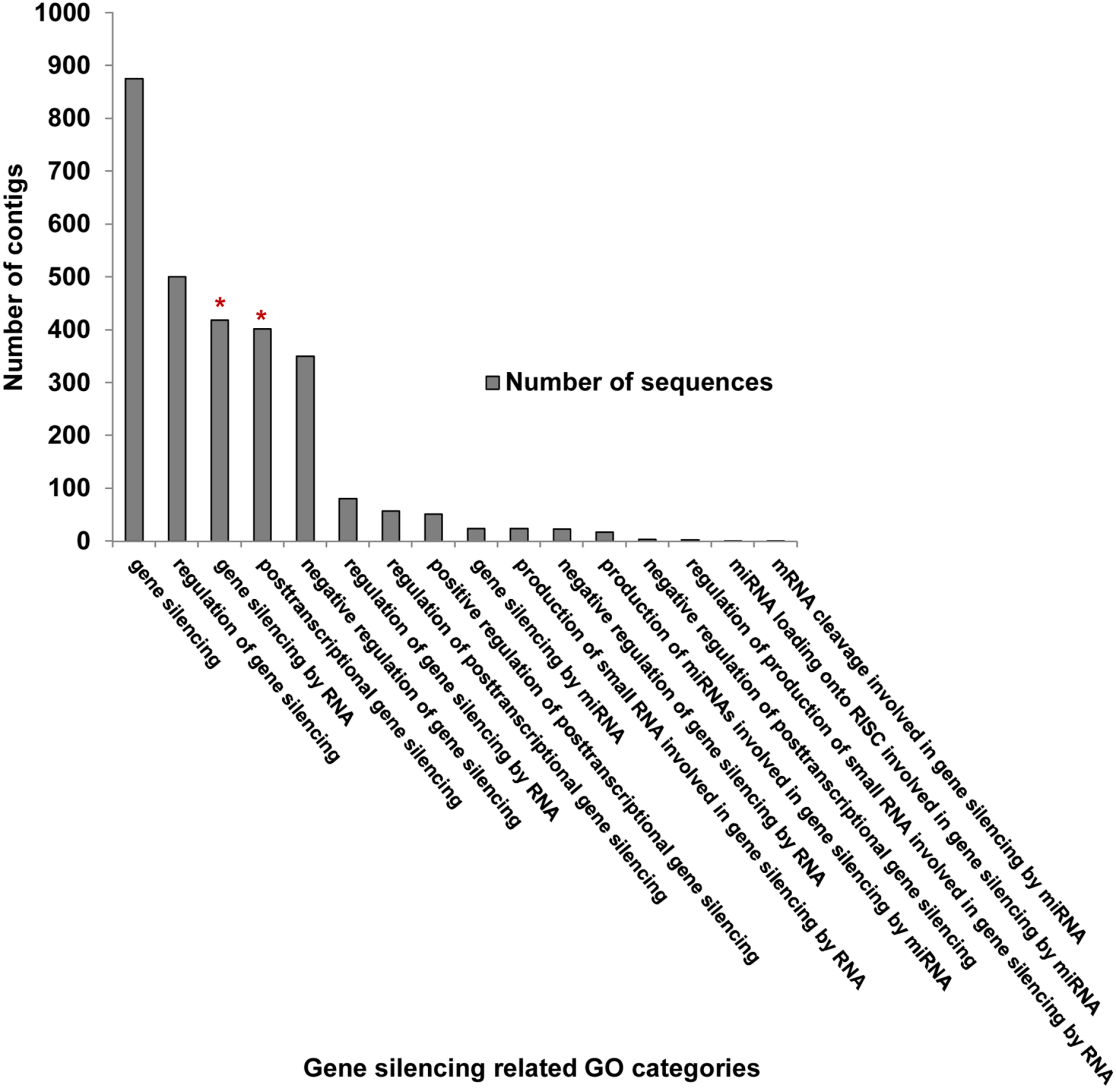
The differentially expressed gene silencing Gene Ontology (GO) categories. GO categories represented on the x-axis were identified using selected search terms, as described in the *Methods* section. The number of ACP contigs was plotted by GO category on the primary axis, shown by the grey bars. Those with significantly different expression levels (*P*<0.05) are noted by a red asterisk above the grey bar. “Differential expression” was based on pairwise comparisons between *Ca*. Liberibacter asiaticus infected and uninfected ACP nymphs and adults.

**Fig 9:**
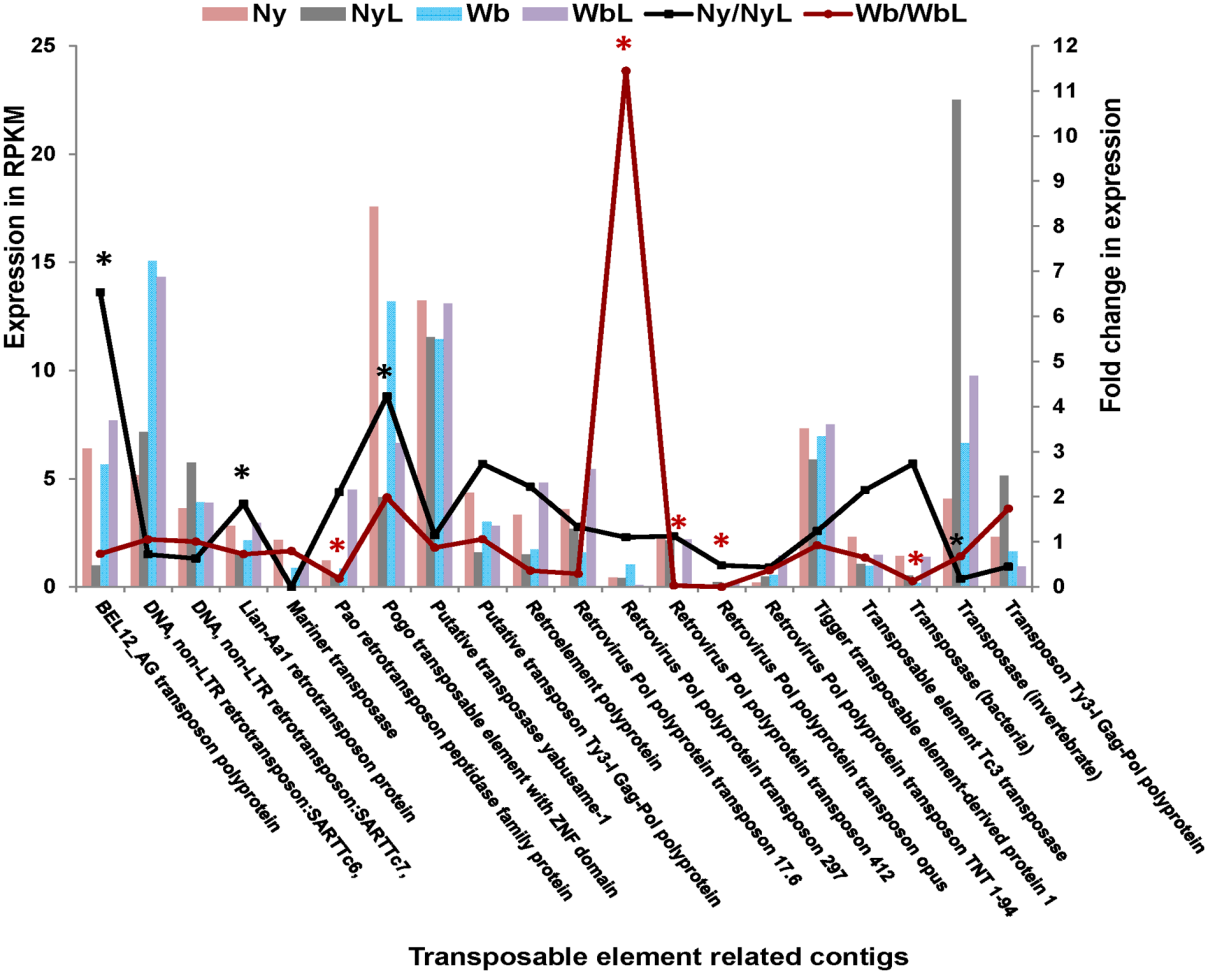
Transposon expression levels, in *Ca*. Liberibacter asiaticus (CLas) infected compared to uninfected Asian citrus psyllid (ACP) adults (Wb x WbL) and nymphs (Ny x NyL). The x- axis shows contigs representing different transposable element categories that were identified using the search terms, as described in the Methods section. Normalized expression in reads, per kilobase per million mapped reads (RPKM) of the transposable elements, is plotted on the primary y-axis for: uninfected nymphal instars (Ny-red bars), infected nymphal instars (NyL- grey bars), uninfected adults (Wb- blue bars), and infected adults (WbL-purple bars). The fold-change response to CLas infection is plotted on the secondary y- axis. The pairwise comparisons between infected and uninfected nymphal instars (NyL/Ny- Black line), and infected and uninfected adults (Wb/WbL-red line), are indicated by the line graphs. Significantly different levels of transcript expression (*P*>0.05) (*asterisk) are shown for infected and uninfected nymphs (black asterisk), and for infected and uninfected adults (red asterisk).

The TCW was used to compile Figs 2, 3, 4, 5, 6, 7, 8 and 9, Tables 1, 2 and 3, and all Supplementary Figures (S1 and S2 Figs) and Tables (S1, S2, S3, S4, S5, S6, S7, S8, and S9 Tables). The TCW Filter Query was used to compile Fig 2 by selecting contigs based on fold change in RPKM value (reads per kilobase, per million mapped reads), an expression measure normalized against library size and length of transcript (in ‘Libraries’), differential expression *p*-values with cutoff limit of 0.05, and annotation status (in ‘Best Hit’). The TCW Filter Query was also used to obtain the contig sequences required to compile Figs 3, 6, and 7 and S1, S2, S6 and S9 Tables by exporting the translated open reading frames of selected contigs. The contigs were mapped to the various biochemical pathways in the KEGG (Kyoto Encyclopedia of Genes and Genomes) Automatic Annotation Server (KASS) using the BBH (bi-directional best hit) method, which implements BLAST comparisons against a set of orthologous groups in KEGG GENES, resulting in KEGG Orthology (KO) assignments and Enzyme Commission (EC) distributions (http://www.genome.jp/kegg/kaas/) [46]. The following three data sets were submitted to KAAS: 1) all of the translated ACP contigs, 2) the translated contigs differentially expressed (p<0.05) between CLas-free nymph (Ny) and CLas-infected nymph comparisons (NyL), and 3) the translated contigs differentially expressed (p<0.05) between CLas-free adult (Wb) and CLas-infected adult comparisons (WbL).

**Table 1:** Summary of DNA sequencing, assembly, and annotation of Asian citrus psyllid transcript libraries.

Sequencing (clean reads)	Wb	21,552,866
	WbL	46,865,913
	Ny	32,265,958
	NyL	28,947,167
	Total	129,631,904
Assembly	Total contigs	45,976
	Mean length (range) (bp)	1,107 (150–26,540)
	% GC (range)	40.4 (10.4–77.9)
Annotation	Total annotated contigs (%)	17,958 (39%)
	Unique hits	293,195
	1st Best hits	9,352
	Total hits	700,796
	Mean length (range) (bp)	1,980 (150–26,540)
	% GC (range)	44.0 (12.3–77.9)
	E-value (range)	7.52E-13 (0–1.0E-13)

Wb = Whole adult body, *Ca*. Liberibacter asiaticus (CLas)-uninfected; WbL = Whole adult body, CLas-infected; Ny = nymphal instars, CLas-uninfected; NyL = nymphal instars, CLas-infected. The consensus transcript sequences are expressed as reads per KB per million reads, or RPKM units.

**Table 2:** Differentially expressed contigs from pairwise comparisons of the *Ca*. Liberibacter asiaticus-uninfected adult and nymphal instars of the Asian citrus psyllid.

Transcript Id	Adult/Nymph (RPKM)	Length	% GC	E-value	Description
DcWN_19757	3493	651	42	1.00E-23	Serine protease easter
DcWN_20608	2103	1132	43.3	4.00E-56	Serine protease easter
DcWN_31964	1543	5286	36.3	8.00E-20	AGAP008244-PA
DcWN_19289	1396	733	43.9	6.00E-22	Cysteine protease 6
DcWN_32183	713	742	62.2	9.00E-65	Uncharacterized protein
DcWN_17954	559	722	42.3	6.00E-13	Vitellogenin-1
DcWN_14150	517	1028	40.8	1.00E-37	Uncharacterized protein
DcWN_30459	474	1660	34.9	3.00E-79	Reverse transcriptase, putative
DcWN_22081	446	491	25.2	5.00E-64	Acetolactate synthase large subunit
DcWN_04909	389	2969	34.6	5.00E-16	Intracellular protein transport protein USO1, putative
DcWN_22191	363	779	41.4	1.00E-22	Protein takeout, putative
DcWN_38201	344	371	24.5	4.00E-17	50S ribosomal protein L32
DcWN_11262	338	3415	34.1	3.00E-22	Bifunctional endo-1,4-beta-xylanase xylA, putative
DcWN_38687	330	430	23	3.00E-59	DNA polymerase III epsilon subunit
DcWN_24273	321	568	45.4	4.00E-17	Putative uncharacterized protein
DcWN_33049	303	228	32.8	1.00E-30	Dihydroxy-acid dehydratase
DcWN_31527	298	254	71.2	3.00E-15	Putative uncharacterized protein
DcWN_07852	295	1774	28.8	0	Mixed type I polyketide synthase-peptide synthetase
DcWN_27346	261	530	29.8	4.00E-52	DNA primase
DcWN_06117	258	1373	42.9	1.00E-37	Hemolymph proteinase 8
DcWN_33624	221	385	23.6	2.00E-38	DNA-directed RNA polymerase subunit beta'
DcWN_25533	220	615	34.1	9.00E-11	S-antigen protein
DcWN_35765	219	311	24.7	7.00E-28	DNA-directed RNA polymerase
DcWN_24274	206	2680	46.7	2.00E-41	LRRC48-like protein
DcWN_16249	199	873	63.2	2.00E-58	Glutathione peroxidase
DcWN_03031	191	4682	31.4	5.00E-44	Viral A-type inclusion protein, putative
DcWN_40242	190	380	56.8	2.00E-27	DnaJ domain containing protein, related
DcWN_16525	179	1305	65.4	9.00E-62	Serine O-acetyltransferase
DcWN_22727	172	985	38.1	5.00E-95	Transferrin
DcWN_16592	149	1466	38.1	5.00E-23	Ubiquitin-protein ligase bre1, putative

**Table 3:** RT-PCR results of 10 selected ACP transcripts usedselected subjected to validation by RT-PCR, cloning, and sequencing of RNA isolated from *Ca*. Liberibacter asiaticus-infected adult psyllids demonstrate the integrity of contigs in the database.

Transcript ID (length bp)^1^	Predicted Annotation (E-value) ^2^	UniProt Id	CLas effect on expression^3^		Primer Sequences 5'-3', forward/reverse
			Nymph	Adult	
DcWN_04644	ABC transporter	E0VTI8_PEDHC	-4.6	-1.6	GCGGGATCCATGACCATCCCTCGAAACCAA
(1947)	(2E-74)				GCGGGGCCCTCACATAGATTTCTTATATTTAAC
DcWN_34339	Sugar transporter 1	C6KIB0_9HEMI	-1.8	-4.4	GCGTCTAGAATGGCGGCAGAGACAAAGAT
(1437)	(3E-69)				GCGGGGCCCTTAGGCGGCAGCGACACCT
DcWN_34586	Putative ferritin 2	Q4PLJ6_ANOGL	1.2	-1.5	GCGTCTAGAATGAATTCAGTACTACTGAATC
(750)	(8E-23)	Q4PLJ6_ANOGL			GCGGGGCCCTTAGGCGGCAGCGACACCT
DcWN_18755	Cathepsin B	A9JSH3_MYZPE	-1.5	-1.4	GCGTCTAGAATGTTGAATCGTCTGGTCCTA
(453)	(2E-106)				GCGGGGCCCGAGATCATCCGATGACAGA
DcWN_16937	Glutathione S-transferase	GST1_BLAGE	-1.1	1.9	GCGTCTAGAATGCCGTCTTACAAGCTGTA
(612)	(2E-21)				GCGGGGCCCTTAGACTTCGGATTTGGGGCGCT
DcWN_13848	Ras-related protein Rac1	RAC1_DROME	-2.2	-2.0	GCGTCTAGAATGCAAGCCATAAAGTGTGT
(589)	(4E-7)				GCGGGGCCCTTACAGGAGCACGCAGCGTTT
DcWN_02176	Mitogen-activated protein kinase kinase kinase A	E2AGG6_CAMFO	-2.8	1.3	GGAAGGTTCGGCAAAGTGTA
(409)	(0)				CGAAGTCTCCCAGCTTCAAA
DcWN_05944	Glycerol-3-phosphate dehydrogenase	GPDM_CAEEL	1.6	-2.6	TGGCCCATTATTGGAAAGAA
(389)	(0)				CGAATGTCGTTGATGGACAC
DcWN_07021	G protein-coupled receptor kinase 2	GPRK2_DROME	-3.7	-1.8	ACTGTTCAACGATTGCATGG
(537)	(0)				CCCAGGTCTGAAATCCTCAC
DcWN_13699	Aquaporin AQPcic	AQP_CICVR	-1.2	1.4	CTCGAAGCAGTCACACCAAA
(307)	(5E-80)				TGCCCAGTAAACCCAAATGT

^1^. Size of amplicon deposited in GenBank [GenBank: KF907310-KF907319].

^2^. Description and annotation of the best hit (E-value -10) from UniProt.

^3^. Numbers represent fold-change in RPKM values. The fold-change ration was determined using TCW.

The TCW Basic GO Query was used to compile Figs 4, 5, and 8 and S3, S4, S7 and S8 Tables, as follows: the histogram (Fig 4) was obtained by selecting for all contigs at GO level 2 and plotting the number of contigs against each category. The significance of the differentially expressed contigs was determined based on *p*-values, using a cutoff limit of 0.05. Significant differentially expressed contigs (*p*<0.05) are marked with an asterisk. S3 and S4 Tables were generated by selecting for categories that were differentially expressed at *p*<10^−10^ for the Ny/Wb, NyL/WbL, and Ny/NyL. The *p*-value cutoff implemented for the Wb/WbL comparisons was less stringent (*p*<10^−5^) to gain a greater range of values for potentially differentially expressed. For the data shown in Fig 5, the database option for up- and down-regulated contigs for the filter query was used to identify the number of up and down regulated contigs using the specific GO ID for each GO category. Also a *p*-value cut off of 0.05 was considered for all queries. The sum of the up- and down-regulated was subtracted from the total number of contigs in each GO category, and the values obtained were expressed as a percentage and are represented on the pie chart for each pairwise library comparison. A ratio ≥1.5 was considered up-regulated, whereas, ratios of ≤ 0.8 were considered to be down-regulated contigs [47]. Fold change values were determined by ‘including’ and ‘excluding’ the specific libraries of interest using the TCW Filter query. In Fig 8, the GO category results for the search term “Gene silencing” were obtained for all contigs at all GO levels with the duplicates removed, and the number of contigs was represented in a histogram. The identification of differentially expressed genes was based on a *p*-value cut off of 0.05, and the significant categories were represented by an asterisk (Fig 8).

The transposon search results (Fig 9) were compiled from the hits that matched the search terms: transposon, TE, retroelement, and transposons. This was done by using the multiple term database search option to maximize the number of hits relating to transposons, because the nomenclature is variable for different groups of transposons depending on the TE class, and whether it is an RNA or DNA TE [48]. Individual contigs that were annotated by the same element were identified, and a single transcript for each was included among the representative contigs reported for each transposon type. Expression levels for transposon hits are shown in histogram for each corresponding library, and fold-change was plotted for comparisons of interest using a line graph and a differential expression *p*-value cut-off of 0.05 with “significance” indicated by an asterisk (Fig 9).

### Assessment of selected psyllid contigs by RT-PCR and DNA sequencing

To assess the overall integrity of psyllid contigs in the TCW database, primers were designed to amplify a subset of contigs using reverse-transcriptase PCR (RT-PCR) for mRNA amplification, followed by cloning and bidirectional DNA sequencing of 3 clones per amplicon (Table 3). The contigs were selected based on predicted biological functions of interest and differential gene expression profiles (*in silico*) in nymphs and adults in response to CLas infection. The sequence for each putative contig was uploaded to FGENESH, a web-based software (http://linux1.softberry.com/berry.phtml) to predict the eukaryotic coding region by homology to other insect reference sequences.

Total RNA was extracted from ACP adults with Trizol according to the manufacturer’s instructions. RT-PCR was performed using the SuperScript III One-Step RT-PCR system and Platinum Taq High Fidelity enzyme. The cDNA synthesis reaction (50 μl volume) contained 25 μl 2X reaction Mix, 10 μM each primer, 2 μl SuperScript III RT/ Platinum Taq, HiFi Mix, 100–200 ng of RNA template, and DEPC-treated water. After cDNA synthesis at 50°C for 30 min, and a denaturation step at 94°C for 5 min, PCR amplification was conducted using 40 cycles of 94°C for 20 s, 53–60°C for 20 s, and 68°C for 1 min, with a final extension at 68°C for 5 min.

The RT-PCR products were visualized by agarose gel electrophoresis in TAE buffer, pH 8.0. RT-PCR products were precipitated by adding 2 X volume of 100% ethanol and 2 μl of glycogen. The precipitated products were washed twice with 70% ethanol, resuspended in TE buffer (10 mM Tris, 1 mM EDTA, pH 8.0), ligated into pGEM-T Easy vector, and transformed into JM109 competent cells. The cloned inserts were verified by colony PCR amplification and DNA sequencing using M13 forward and reverse primers. The colony PCR products were purified with the GeneJET plasmid Miniprep Kit (Fermentas) according to the manufacturer’s instructions, and DNA sequencing was carried out at the University of Arizona Genetics Core Facility (Tucson, AZ USA). A consensus transcript (contig) sequence was submitted to the GenBank database as the Accession numbers KF907310-KF907319.

## Results

### Illumina sequencing, assembly and annotation

Polyadenylated ACP RNA was isolated and used to construct four Illumina paired-end sequencing libraries, from pools of CLas-free whole adult bodies (Wb) or nymphs (Ny) (instar stages 2–5) or from CLas-infected adult (WbL) or nymph (NyL) psyllids. Sequencing was carried out as described in the Methods and produced 129,631,904 clean reads total, with 21,552,866 for Wb, 46,865,913 for WbL, 32,265,958 for Ny, and 28,947,167 for NyL. Reads were assembled, yielding 45,976 contiguous sequences (contigs), which represent potential transcripts. The average GC content of the contigs was 44.4% (range of 10.4–77.9%) with the contig length ranging from 150 to 26,540 bp (87% > 200 bp, 57% >500 bp, and 37% >1000 bp; S1 Fig). Reads were mapped back to the contigs to compute the quantitative expression level for each. The average number of reads mapped to contigs was 2,819, with an average of 149 reads per RPKM for the combined libraries. Of the 45,976 contigs, a relatively small number were found to be uniquely present in only one library. These included 55 contigs in Wb, 80 in Ny, 206 in WbL, and 117 NyL (Table 1).

The contigs were annotated based on comparison to the UniProt invertebrate, bacterial, and virus databases with 10^−10^ as the BLAST cutoff. Thirty-nine percent (17,598) of contigs had at least one hit. The average E-value was 7.52×10^−13^ and 98% of the best hits to ACP contigs were other invertebrate sequences.

Approximately 26% of the ACP invertebrate-associated contigs shared greatest homology equally with the pea aphid, *Acyrthosiphon pisum* (Harris) Order: Hemiptera and the human body louse *Pediculus humanus capitis* (L.) genome sequence Order: Phthiraptera (S2 Fig). Similar results were reported for annotated potato psyllid transcripts [33]. The remaining 2% of annotated contigs shared greatest homology with bacterial sequences for two previously identified endosymbionts of psyllids, *Ca*. Carsonella ruddii [49] and *Wolbachia*, [50], and with other insect viruses that shared high sequence identity to baculoviruses and transposable elements.

### Differential expression, Kyoto Encyclopedia of Genes and Genomes (KEGG) and Gene Ontology (GO) analyses

The TCW was used to identify psyllid genes that were differentially expressed for ACP harboring or free of CLas. The analyses were based on pairwise comparisons of within-and-between differences among transcript expression levels for each library-treatment combination, CLas-infected compared to-uninfected nymph or adult stage ACP. Approximately 36% of the total contigs were differentially expressed, and of these 14% were annotatable (Fig 2A). Among the annotated contigs the majority of differential expression was 10-fold or more for all possible treatment comparisons e.g. life stage (nymphs and adults) and CLas presence/absence (Fig 2B).

Using KEGG pathway analyses software and databases, which predict and map biological-functional interrelationships between genes, the ACP contig assignments spanned 330 pathways [46]. Of these 17% (55) and 83% (275) were associated with metabolic and non-metabolic pathways, respectively (S1 Table). Purine, carbon, pyrimidine, glycerophospholipid, and choline metabolism were among the top KEGG metabolic-associated pathways. Among the non-metabolic pathways the top 10 reflected disease- and viral-infection associated pathways, and endocytosis. The *Endocytosis* pathway is of interest because it is used by other pathogens for host invasion [33, 51]. Of the 76 psyllid contigs identified in this pathway, eighteen and 7 were significantly (p<0.05) differentially expressed in response to CLas in ACP nymphs and adults, respectively (Fig 3, S2 Table). This result may reflect the requirement for CLas invasion of first the nymph and then the adult stage, respectively, in the propagative, circulative life (infection) cycle in the psyllid host. These results suggest that the host genes are more highly targeted by CLas during invasion of the immature instars compared to adult psyllids. Further, clathrin, a protein involved in the selective internalization of molecules during receptor-mediated endocytosis [52], was significantly down-regulated in both the nymphal and adult life stages. In RNAi knockdown experiments, reduced clathrin expression resulted in a 5-fold decrease in invasion of human epithelial cells by *E*. *coli* and *S*. *aureus* [53]. By analogy ACP may modify its gene expression as a defense against CLas invasion.

The classification of ACP contigs was based on GO categories (Fig 4). The level-1 GO distribution was highest for the *Biological Processes* with 57.5% of transcripts, followed by *Cellular Components* with 24.5%, and *Molecular Function* with 18%. The level-2 GO assignments were dispersed among 46 GO categories.

The majority of transcripts assigned to *Biological Processes* GO category were distributed among the *Biological Regulation*, *Cellular Processes*, *Developmental Processes*, and *Metabolic Processes* (Fig 4). The *Cellular Component* GO category contained transcripts in the sub-categories: *Cell*, *Macromolecular Complex*, *Membrane*, and *Organelle*. Most of the transcripts in the *Binding and Catalytic Activity* category contained transcripts representing *Molecular Function*. The collective results mirror the dynamic lifestyle of ACP, and its capacity to adapt to different environments. Categories that were significant as far as differential expression among different treatments is concerned are discussed further.

Perhaps unexpectedly, the comparative transcript profiles suggested that psyllids generally tolerate CLas-infection, and is based on the observation that most ACP transcripts were not significantly differentially expressed (Fig 5; *p*<0.05; fold change ratios of < 2 are considered unchanged). Such a pattern suggests that ACP adults and nymphs are relatively well adapted to CLas infection and perhaps that this symbiotic relationship is evolutionarily longstanding. Even so, the ACP nymphs showed more changes in gene expression than the adults (Fig 5), suggesting that the early psyllid life-stages are critical targets for invasion, multiplication, circulation, and perhaps salivary gland-mediated acquisition. This also could suggest that adults have evolved reasonably high tolerance to CLas infection owing to a long-standing host-parasite relationship [2, 21]. One possibility is that CLas primarily exploits the nymphal over the adult stages, to invade and establish systemically and to a high titer, during vulnerable but highly metabolically active stages of growth and development. In this way it would already occupy surviving adult organs and perhaps be able to down-regulate pathogenesis-related activities during psyllid adulthood to ensure the reproduction of and transmission by its psyllid host, both which would be undermined if resources were depleted.

It is well known that ACP adults reared on CLas-free plants are not competent vectors of the bacterium to citrus plants, even when given acquisition-access to CLas [18]. Based on these results, adults may be entirely incapable of transmitting CLas unless they ingest and/or acquire it during the late nymphal and/or early adult stages. Taken together with the results reported herein, we hypothesize that adult-mediated CLas transmission depends on nymphal infection with acquisition occurring in the adult stage, in order to achieve a titer threshold sufficient to invade late-nymphal and/or adult psyllid salivary glands to assure CLas inoculum to be transmitted the plant host by its vector. Perhaps when these or other scenarios are borne out through additional studies, the higher number of significantly up- and down-regulated contigs observed here in response to CLas infection of nymphs (Fig 5A–5C) over adults (Fig 5D–5F) will be more definitive. Even so, the particular groups of differentially expressed transcripts already offer interesting clues that, at least in part, support the above hypotheses, in that among the major GO categories were *Biological Process* (28% in adults and 39% in nymphs, respectively), *Cellular Component* (27% in adults and 40% in nymphs, respectively) and *Molecular Function* (28% in adults and 39% in nymphs, respectively). The significant down-regulation of immunity and defense genes, observed particularly in the nymphal stages, could possibly render ACP nymphs more permissive (susceptible) than adults to CLas invasion and multiplication, thereby facilitating CLas spread into key anatomical sites in the late-stage nymph and/or early adult stage such that acquisition occurs by the time young adults become mobile, reproduce, and transmit the bacterium to the flush growth of the host plant.

### Contigs associated with differences between adult and nymph developmental stages

The contigs having the greatest differential expression in the CLas-free adults and nymphs (based on RPKM values) represent genes utilized in behavioral responses, cell differentiation, development, and growth (Table 2), and include acetolactate synthase, apyrase, arginine kinase, arylsulfatase, cysteine proteinase, glutathione peroxidase, polyketide synthase, serine protease ester, transferrin, and vitellogenin. For example, arginine kinase expression was upregulated by ~70-fold in adults compared to nymphs, and perhaps is not surprising because it is an enzyme involved in cellular energy metabolism [54]. This is consistent with the adult stage requirement for maximum energy essential for reproduction dispersal, and survival during shortages of food, whereas, nymphs are able to feed immediately and consistently for the most part where they are borne.

The ACP nymphal genes found to be overexpressed by greater than 10-fold were calmodulin, hedgehog, survivin, talin 2 and wnt. Such genes are known to be activated during energy-intensive processes including development, cell and tissue differentiation, growth, and molting in nymphs [55–58]. In addition to wnt, twenty-one other contigs were identified in the *Hedgehog signaling* pathway, constituting 85% of the predicted pathway members (S1 Table). Additionally, arylphorin-type storage protein, acyl-CoA delta-11 desaturase, lazarillo protein, and a cuticle protein were up-regulated in nymphs. In particular insect cuticle proteins are temporally regulated, and are up-regulated during tissue-specific cell differentiation during molting and metamorphosis [59]. Arylphorin storage proteins are ubiquitous in insects, have high aromatic amino acid content, and support growth and development, particularly in larval hemolymph-defense responses [60]. And, acyl-CoA delta-11 desaturase catalyzes the formation of delta (11) fatty acyl precursors for phospholipid membrane incorporation and modulation of membrane fluidity, a primary adaptive cellular response [61].

Overall, the gene expression patterns for nymph and adult comparisons reflected distinct global patterns of protein and energy requirements of both the immature and adult life stages, whereas, expression level comparisons between CLas-uninfected and—infected psyllids underscored infection-specific genes. Of particular interest for RNA-interference (silencing) objectives, therefore, are genes involved in initial CLas invasion of the psyllid gut, gut establishment, systemic spread into and throughout the hemolymph and other organs, and for CLas entry and/or proliferation in the salivary glands.

### Contigs of interest with altered expression in CLas-infected ACP nymphs

In early nymphal stage(s) (1–3) following CLas infection (not taking into account possible transovarial transmission), expression profiles (herein) and preliminary results of electron microscopic analyses (authors, unpublished), the bacterium appears to have established and persisted in most tissues and organs. Examples of CLas effectors alluded to based on the expression profiles were candidates with likely (putative) involvement in invasion and pathogenicity. Among them were proteins essential for cellular adhesion, biofilm formation, motility, and circulation in the hemolymph, presumably *en route* to the oral region/salivary glands [8, 9]. Also, genes involved in development, morphogenesis, and innate immunity were prominently overexpressed in the nymphal instars (S3 and S4 Tables); results that strongly suggested CLas is capable of suppressing basal immunity, which could modulate nymphal development and affect immature stadia longevity in ways that are beneficial to CLas invasion, multiplication, and circulation during early stage infection of its psyllid host.

#### Adhesion/Invasion-related contigs

Consistent with the circulative, propagative transmission hypothesis, laminin isoforms were upregulated in CLas-infected compared to the uninfected ACP nymphs. Because laminins are essential for dermal membrane formation during development [62], their up-regulation could represent a response to membrane formation disruption by CLas while entering or exiting cell, tissue, or organellar membranes.

In addition, vinculin was down-regulated in the infected nymphs. Vinculin links the actin cytoskeleton to adhesion receptors in focal adhesions and adherence junctions [63]. Talin, a protein known to contain vinculin binding sites [64] was also down-regulated in infected nymphs. Interestingly, the *Rap 1 signaling* pathway, which involves talin, and regulates focal adhesion dynamics (Fig 6), was among the top 25 non-metabolic KEGG pathways (S1 Table). During invasion *Shigella* species modulates host vinculin expression to promote infection [65], making its down-regulation potentially important in CLas invasion processes. Certain *Rickettsia* spp. express a talin mimic to gain entry into the host cell [66]. These results suggest that both vinculin and talin could be important for CLas invasion. Further, down-regulation of psyllid gene expression in such inter-related pathways provides important clues to the effect that CLas appears to modulate cytoskeleton networks to gain access to intercellular and intracellular space in its host, while also enhancing its persistence.

#### Defense/Immune response-related contigs

A matrix protein gene required for wound healing, tenascin [67], and semaphorin, known to be involved in axon guidance and immunity in insects [68] were both down-regulated in infected ACP nymphs (S5 Table). Wound healing in psyllid organs at sites of bacterial entry or exit could restrict CLas to particular areas, and if so, the CLas-mediated interference with wound-healing proteins would hold open and make accessible such sites. Further, the expression of cathepsin B, a protease-like enzyme utilized during molting and stress in insects [69], was reduced in infected nymphs (Table 3) potentially giving CLas a positive advantage if ACP-encoded proteolytic enzymes are used by the host to defend against CLas invasion. An ABC transporter-like gene was also down-regulated in ACP nymphs (*p*<0.01, see Table 3). ABC transporters are integral membrane proteins that transport solutes across membranes via ATP hydrolysis whereas others are involved in maintenance or RNA and DNA repair in the cytosol [70]. Mutations in an ABC transporter of *Heliothis virescens* were associated with its resistance to attack by the bacterial pathogen *Bacillus thuringiensis* (Bt) [71] by preventing Bt toxin binding to the membrane. Down-regulation of ABC transporters in infected nymphs may be regulated by CLas to gain advantage by interfering with host membrane transport, or the host itself may have responded by counter attack, perhaps to undermine the transport of damaging CLas-imposed compounds such as anti-bacterial peptides.

#### Nutrition-related contigs

Chitinase was up-regulated in CLas-infected nymphs (S5 Table). Many bacteria, including *Listeria monocytogenes* and *Vibrio cholerae*, which are animal pathogens [72, 73], and *Xylella fastidiosa*, a phytopathogenic bacterium transmitted by certain planthoppers in a non-circular manner [74], hydrolyze and utilize host chitin for a nutrition. Chitinases also enhance bacterial virulence by suppressing host innate immunity [73]. Accordingly, Liberibacter effectors may interact with or stimulate these and other enzymatic activities during infection to facilitate CLas access to psyllid organs.

Apolipophorin is a protein required for dietary lipid transport and required for insect flight [75] and also has been associated with virulence of certain pathogens. In the silkworm, apolipophorins inhibit hemolysin expression in *Staphylococcus aureus* to combat bacterial virulence [76]. Apolipophorin expression in CLas-infected nymphs was down-regulated (S5 Table), indicating that CLas could affect lipid transport, thereby reducing the availability of dietary lipids essential for membrane synthesis, thus rendering nymphs susceptible to CLas invasion.

Transferrin, a glycoprotein required for iron transport [77], is recognized by bacterial outer membrane receptors of transferrin-iron complexes, which internalize iron without the use of siderophores [78], was down-regulated in CLas-infected nymphs (Table 2). Also, transferrin is a recognized as a virulence determinant for *Wuchereria bancroftti*, the causal agent of elephantiasis, which is transmitted by the *Aedes aegyptti* in a circulative-propagative manner [79]. These observations suggest that CLas alters the local host environment such that nutrients are more freely available for its use. Also, ACP expression of ferritin, a storage protein for non-toxic forms of iron [80], was up-regulated in infected nymphs (Table 3), suggesting that they sequester free forms of iron, which could lead to iron starvation of CLas as a means of combatting nymphal infection.

### Contigs of interest with altered expression in CLas-infected ACP adults

After the last molt e.g. from the 5th nymphal instar to adult, the young adults (tenerals) are not yet mature. Within several days tenerals mature into adults and the physiological processes shift to storing carbohydrates and fats for dispersal and reproduction. The nutritional needs of adults differ from those of nymphs, which typically remain associated with the plant on which they were born. Adult psyllids must survive hardships and environmental stresses during dispersal and reproduction, and this is dependent upon the overall health of young adults that aids them in locating nutritionally optimal food sources. Expression profiles provided a strong indication that the defense and immune response genes of CLas-infected adults were more robustly expressed compared to the nymphs, even though all stages are infected. This suggests that ACP adults, over nymphs, have a more robust immune system against CLas, either because adults are better able to overcome infection pressures, and/or that perhaps late in the infection cycle CLas does not strongly modulate adult immunity, or both.

#### Adhesion/Invasion-related contigs

More than 1,700 contigs were assigned to the *Biological Adhesion* and *Extracellular Matrix* categories (Fig 4) and are of interest because of their possible roles in supporting the propagative, circulative relationship of CLas with its host. The accumulation of CLas occurs in regions of the CLas-infected alimentary canal [8, 9] and lesions apparent on the external surface through which bacteria appear to exit. Thus, CLas infection requires bacterial adhesion to psyllid membranes and other surfaces, and that it interacts with the cellular matrix and membrane proteins. Among the differentially expressed genes, a protein involved in basal membrane formation, papilin, was down-regulated in CLas-infected adults (S5 Table). In *C*. *elegans*, the suppression of papilin expression was lethal to the embryos owing to the disruption of basement membrane formation [81]. Thus the down-regulation of papilin-like transcripts in CLas-infected psyllid adults suggests this protein may be utilized by CLas during initial invasion and possibly systemic spread in the psyllid. Also, several additional proteins with potential involvement in CLas invasion of ACP were differentially expressed (S5 Table) including integrin, a type of cell adhesion molecule [82] involved in innate immunity, echinoid that functions in cell adhesion and cell sorting [83], and fibrillins, which are structural proteins integral to microfibrils in tissues and muscles [84]. The basis for CLas-mediated down-regulation of the expression of these particular genes is unknown, however, (putatively) weakened muscles and other tissues known to facilitate adhesion and infection processes may provide an explanation.

#### Defense/Immune response-related contigs

Many differentially expressed genes were assigned to the GO categories: *Antibiotic Biosynthetic Process*, *Encapsulation of Foreign Target*, *Extracellular Matrix*, *Melanization Defense Response*, and *Response to Topologically Incorrect Protein*, which are commonly involved in defense responses. For example, phenoloxidase, which is required for melanization [85] was down-regulated (S5 Table) suggesting CLas suppression of the ACP immune system. Also, hemocytin, a blood protein involved insect humoral responses and that shares homology with the mammalian von-Willebrand factor of like function [86] was down-regulated in CLas-infected adults (S5 Table). It is possible that CLas effectors interact with hemocytin to modulate circulation in psyllid hemolymph to systemically infect the host.

Rac1, a member of the Rho family of GTPases that operate as molecular switches to regulate immunity, actin dynamics, gene transcription, and cell cycle progression [87], was up-regulated (Table 3, Fig 6, S6 Table) in CLas-infected adults. In other insects attacked by parasitoids and pathogens, Rac1 up-regulation [88] has been linked to hemocyte recruitment [89, 90]. Expression of Jun N-terminus kinase (JNK)-like and mitogen-activated protein kinase kinase (MAKK)-like genes (Table 3) were up-regulated in ACP. These proteins act in signaling pathways that regulate apoptosis, cell proliferation, differentiation, migration, and stress response, and are activated by Rac-1(Fig 6) [91], signaling activation of the immune system to counter CLas invasion.

Finally, a number of immune-related contigs (S7 Table) shared similarities with those identified previously in the pea aphid [92, 93], which has a relatively ‘limited’ immune system. It has been suggested that the innate immune system has evolved in a way so as to reduce attack on the primary and secondary endosymbionts that provide essential amino acids, hormones, and nutrients [92–94]. In contrast, honeybee larvae able to express a complete repertoire of immune proteins were unable to defend themselves against the bee pathogen *Paenibacillus larvae* even though during the adult stages bees were resistant to bacterial infection [95]. Thus despite a robust immune system all stages do not counter pathogen attack equally.

#### Nutrition-related contigs

Expression analysis identified 14 ACP contigs assigned to the *Nutrient Reservoir Activity* GO category (Fig 4). These are of potential interest because of their involvement in nutritional support for CLas establishment, invasion of the gut, hemolymph. For example, hexamerin, an amino acid storage protein [96], was down-regulated in CLas-infected adults (S5 Table) suggesting that CLas may modulate free amino acid availability by interfering with hexamerin storage pathways by regulating expression of amino acid storage protein genes.

Vitellogenin-1 expression also was responsive to CLas infection of ACP (Table 2). Vitellogenins are glycoproteins that serve as a rich source of nutrients, are a major egg protein, and are up-regulated in some insects in response to stress [97]. In ACP, vitellogenin was down-regulated unexpectedly, suggesting that CLas may deprive ACP of this protein. Although somewhat speculative, this is supported by evidence of reduced oviposition and fecundity in the related Liberibacter-potato psyllid pathosystem [98], and is consistent with pathogen-induced host manipulation known in somewhat analogous systems, including the *Anopheles*-*Plasmodium* and *Tenebrio molitor* beetle-tapeworm complexes [99, 100].

### Differential impact of CLas infection on immature and adult psyllids

Comparisons of expression profiles of the adult and nymph ACP life stages revealed stage-specific differences and new insights into CLas invasion tactics, particularly involving defense responses (S5 Table). Among these were aldo-keto reductase, chorion peroxidase, and intracellular protein transport protein USO1 [101, 102].

#### Gene silencing-related contigs

In other insect defense systems, immune deficiency (IMD) and Toll pathways are known to combat fungal and bacterial pathogens, whereas, the RNA interference (RNAi) pathways are implemented primarily to combat viruses [103]. Also, microRNAs (miRNAs) or small RNA molecules associated with insect RNAi-antiviral pathways [104] are expressed during *Wolbachia* infection of the mosquito *Aedes agypti* to strategically modulate gene expression of its host [28]. The *MicroRNAs in cancer* pathway was among the top 40 non-metabolic KEGG pathways (S1 Table). Also, several contigs involved in controlling gene expression, post-transcriptionally, by the *RNA degradation* pathway were differentially expressed in CLas-infected nymphs and adults (Fig 7).

Importantly, contigs assigned to RNAi-related GO functional categories (Fig 8) were found to be present, making this the first report of RNAi machinery in ACP. Expression levels of RNAi-related transcripts were down-regulated to a greater extent in CLas-infected nymphs compared to adults (S8 Table). Also, the antiviral helicase Ski2, a gene involved RNA degradation (Fig 7, S9 Table), including decay of mRNA targeted by the RISC complex [105], was up- and down-regulated in nymphs and adults, respectively, indicating life stage differences in post-transcriptional processing. Even though RNAi machinery is active in both adults and nymphs, whether it modulates CLas invasion of one or both life stages as a pre-requisite for adult-mediated transmission [18], is unclear.

Genes sharing similarity to *regulator of the nonsense transcript 1* homolog, PIWI, and SID1-like proteins were down-regulated in nymphs compared to adults. Potentially, knock-down of PIWI, a component of the PIWI-interacting RNA (piRNA) pathway involved in transposon silencing, may result in genome instability mediated by transposable element (TE) de-repression [106]. The expression levels of DXX6, an ATP dependent RNA helicase component of the *RNA degradation* pathway (Fig 7, S9 Table) having a role in piRNA biogenesis [106] was down-regulated in CLas-infected nymphs. Also, the presence of SID1 transcripts, which encode a putative transmembrane protein essential for systemic RNAi, indicates that ACP has such a systemic pathway [107], which has also been reported in certain aphids [108] and several other insects. In contrast there is evidence that dipterans such as *Drosophila melanogaster* or *Anopheles gambiense* utilize a cell-autonomous RNAi pathway [109, 110]. ACP contigs annotated as Dicer and Ago3, other well-known participants in the RNAi pathway [111], were not down-regulated in CLas-infected nymphs or adults. Interestingly, in *Drosophila* viral suppressors are known to target the host silencing machinery and suppress the RNAi silencing-mediated immune response [112]. However, in ACP, even though phage and other viral sequences are represented in the transcriptome, there was no evidence of CLas-associated effector stimulation of this suppression mechanism.

Certain viral proteins commonly stimulate stress responses that upregulate gene silencing pathways in the host. For example, a *nemo*-like transcript has been attributed to genotoxic responsiveness and regulation of innate immune responses against RNA viruses [113, 114]. Transcript profiles showed a *nemo*-like transcript down-regulated in CLas-infected nymphs (S5 Table) suggesting involvement of transposon or phage activation and is supported by the observed differential expression of genes annotated as *virus-like* in the ACP transcriptome.

#### Transposon-related contigs

The CLas genome encodes non-transport ABC proteins that are expected to regulate gene expression, repair DNA and RNA, and excise transposons [115]. In *A*. *gambiae* [116] and *A*. *aegypti* [117] transposable elements (TEs) remodel eukaryote genomes through rearrangements [118] and alter different functions in the host, some which are known to affect environmental adaptation [119]. TE insertions in *Culex quinquefasciatus* mosquito have been associated with insecticide resistance [120]. Evidence for transposon reactivation-associated contigs in the ACP transcriptome may provide evidence for altered genome architecture associated with ‘environmental’ adaptation. If so, these and other epigenetic signatures could explain in part the evolution of different ACP haplotypes or strains [121, 122]. Given that TE invasion would be expected to result in concomitant disruption of genome integrity, expression level changes in the putatively TE-associated contigs was investigated (Fig 9). Indeed, a number of differentially expressed TE contigs were identified that could provide support for mobile element activity to be related to CLas-infection.

The expression of TE, *Wolbachia* and *C*. *ruddii*-like transcripts supports our contention that a complex host-parasite/pathogen-microbiome (bacterial and viral) community and its multi-partite, *trans*-kingdom interactions are essential for CLas systemic invasion of ACP that leads to circulative, propagative transmission. As such, there appears to be great potential for disrupting effectors encoded either by CLas and/or its associated microbial community, notwithstanding-virus-like effectors, and exploiting them as biopesticide targets to abate the ACP-mediated CLas transmission pathway.

## Discussion

The on-line ACP annotated transcript database (http://www.sohomoptera.org/ACPPoP) has been established and interfaced with the easy-to-use Transcriptome Computational Workbench (TCW) tool, providing the first comprehensive computational resource for parsing differentially expressed genes of interest with high statistical significance.

The ACP transcript profiles were mined using TCW and KEGG pathway mapping to identify candidate psyllid proteins with predicted involvement in CLas-effector interactions, leading to invasion, multiplication, and circulation in the vector. Among the candidate transcripts were genes with predicted involvement in nutrition, and immune system and defense responses. Additionally, transcripts involved in gene silencing and TE activity were classified in biologically significant categories associated with CLas invasion and long-term host fitness that strongly implicate the activation of gene silencing pathways and TE accumulation [123]. Evidence of TE element activity is particularly intriguing owing to potential roles they may have in CLas pathogenesis, genome architecture evolution, and epigenetic inheritance.

The *in silico* comparative analyses of contigs in the CLas-infected and—uninfected nymph and adult stages showed many with predicted involvement in CLas invasion, adhesion, multiplication, biofilm formation, and nutritional parasitism. The results are striking in that they reveal differential responses by ACP life stages that are in line with CLas pathogenicity, namely, the (apparent) greater susceptibility of the ACP nymphal, over the adult, stages to CLas-infection. This proposed requisite for early-ACP stage CLas invasion, followed by an apparently, lowered virulence i.e. psyllid counter attack or CLas lessened pathogenicity might be explained by the need to minimize adult damage, which if excessive could impede adult dispersal, feeding, and reproduction, activities that promote ACP-mediated CLas transmission to the plant host. In this scenario, CLas manipulates the immature ACP host stages to ensure multiplication and attainment of a high titer to optimize circulation in the hemolymph and localization in the salivary glands. This is further supported by the observation that CLas does not interrupt essential developmental processes of the nymphal stages to the extent that growth, molting, or sexual maturation ultimately proceeds to completion. However, based on contigs assigned to a number of the major GO categories, certain processes appear to have been delayed temporarily. Even so, different expression profiling using both TCW annotations and KEGG pathway analyses suggest that CLas suppresses the immune system of both nymphal and adult stages. Taken together, ACP nymphs and adults both appear to provide distinct, essential contributions that offer key vantage points to CLas during psyllid host infection and systemic spread.

The *in silico* hypotheses presented here set the stage for new challenges to achieve next steps. Biologically relevant ACP proteins will require functional confirmation of protein-protein interactions with counterpart Liberibacter effector(s), and through direct knockdown using RNAi. From this study, a number of lucrative genes show promise as targets for abatement of psyllid-mediated CLas systemic invasion of ACP in the circulative, propagative transmission pathway leading to reduced transmission competency. Much additional information is still needed to elucidate the CLas transmission pathway, and to identify the most lucrative effectors that mediate the navigation of CLas through its (putative) secondary host. This will require a high level of understanding of the diverse array of proteins and other molecules that mediate pathogenesis/parasitism processes in this novel multi-partite, trans-kingdom community.

Finally, results demonstrate the presence of TE elements, and open the door for potentially harnessing them to drive gene expression in ACP using expression cassettes that can be engineered for psyllid transformation. Similarly, insertional mutagenesis and P-element insertions have been used in *Drosophila* to investigate gene function and genomic evolution. Transformation of psyllids with active TEs such as *hobo*, *mariner*, *minos*, or *piggyback* could aid in the transgenesis of psyllid lines having the potential for use in gene or gene-enhancer trapping, or possibly genome-wide insertional mutagenesis [124–127].

Powerful functional genomics and genetics approaches now promise unique opportunities for exploiting vulnerabilities in the CLas circulative-propagative pathway that could have direct bearing on CLas invasion and parasitism of ACP by interfering with key processes involved. Of particular interest for abatement of the infection-transmission cycles are ACP proteins required by CLas invasion-virulence, biofilm formation, and systemic circulation, and for salivary gland localization and acquisition targets. Those demonstrated to result in gene silencing mediated dsRNA, and delivered to the host plant for ingestion by psyllid, and/or through genetic modification of the psyllid itself, will offer new, versatile prospects for HLB management. Such novel biopesticide technologies have the potential to reduce or knock-out altogether CLas survival during gut invasion or multiplication, prevent its entry into and/or circulation in the hemolymph, or impede (putative) receptor-mediated entry into the salivary gland and/or vector acquisition itself. In short, achieving a CLas-diminished state within the ACP host has great potential to ‘replace’ the extant CLas-infected ACP populations with CLas-free psyllids, and thereby abate both horizontal and vertical transmission of the HLB pathogen between its primary and secondary hosts.

## Supporting Information

S1 Fig The distribution of contig lengths in the assembled transcriptome of the Asian citrus psyllid (*Diaphorina citri*) for the annotated and unannotated contigs. Of the 45,796 unique contigs (blue bars), 18,901 were annotatable (red bars) using reference sequences available in all publicly available UniProt databases. The majority of annotated contigs were 1–2 kb in size, with the number of contigs shown on the Y-axis, and the size range of contigs in base pairs represented on the X-axis.
(TIF)

S2 Fig The distribution of the ACP Wb/WbL and Ny/NyL annotated contigs for which sequences are available in the SwissProt and TrEMBL invertebrate databases. Insects comprised the top ten invertebrate species that enabled annotation of the ACP transcriptome.
(TIF)

S1 Table List of KEGG pathway assignments for all differentially expressed (*p*<0.05) contigs. (CSV)

S2 Table List of differentially expressed (*p*<0.05) contigs assigned to the endocytosis KEGG pathway. (CSV)

S3 Table The Gene Ontology categories having a significant number of differentially expressed contigs (*p*<10^−10^), based on pairwise comparisons between *Ca*. Liberibacter asiaticus (CLas)- uninfected and-infected Asian citrus psyllid nymphal instars and adults, Ny x Wb, NyL x WbL, respectively. (CSV)

S4 Table The Gene Ontology categories of the pairwise comparisons for differentially expressed contigs for (a) *Ca*. Liberibacter asiaticus (CLas)-uninfected- and CLas-infected nymphal instars (NyNyL; *p*<10^−10^), and (b) CLas-uninfected and CLas-infected adults (WbWbL; *p*<10^−5^) of the Asian citrus psyllids.
(CSV)

S5 Table List and summary of statistics for all Asian citrus psyllid transcripts, with the normalized expression values (RPKM), differential expressional values (*p*-value), UniProt annotated descriptions, and taxonomic database source of annotation. (CSV)

S6 Table List of differentially expressed (*p*<0.05) contigs assigned to the *Rap 1 signaling* KEGG pathway. (CSV)

S7 Table Total number of Asian citrus psyllid (ACP) transcripts assigned to immune system-related GO categories (a). A list of all ACP transcripts assigned to the Immune deficiency (b) and Toll (c) signaling pathways.
(CSV)

S8 Table List and summary of statistics for the RNA interference (RNAi) pathway-associated Asian citrus psyllid transcripts including normalized expression values (RPKM) and UniProt annotated descriptions. (CSV)

S9 Table List of differentially expressed (*p*<0.05) contigs assigned to the *RNA degradation* KEGG pathway. (CSV)
