# Supplementary figures and images for: Asian Citrus Psyllid Expression Profiles Suggest Candidatus Liberibacter Asiaticus-Mediated Alteration of Adult Nutrition and Metabolism, and of Nymphal Development and Immunity
Source: PLoS One. 2015 Jun 19;10(6):e0130328. doi: 10.1371/journal.pone.0130328 (PMC4474670; doi:10.1371/journal.pone.0130328)

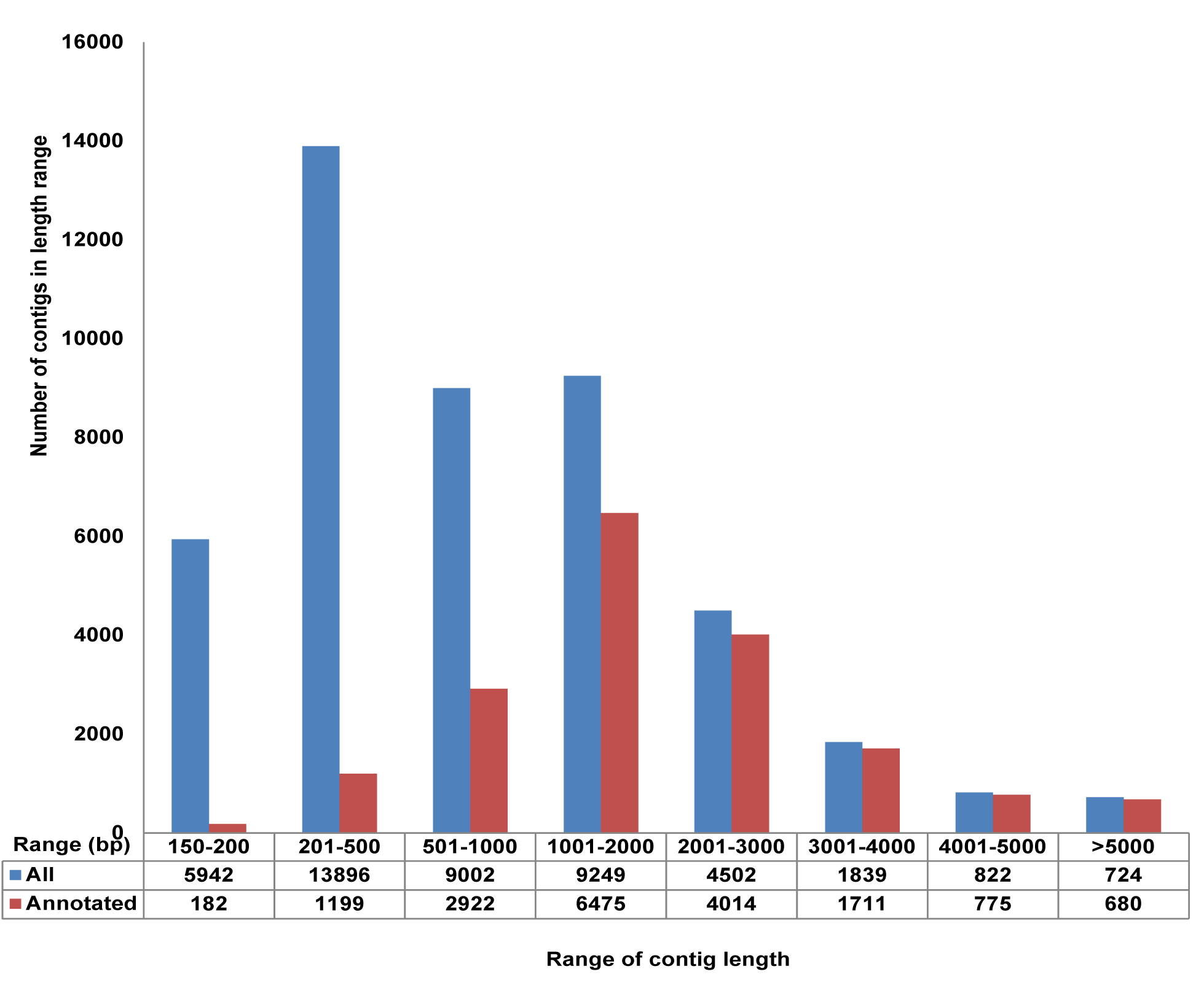

Supplement: S1 Fig — Of the 45,796 unique contigs (blue bars), 18,901 were annotatable (red bars) using reference sequences available in all publicly available UniProt databases. The majority of annotated contigs were 1–2 kb in size, with the number of contigs shown on the Y-axis, and the size range of contigs in base pairs represented on the X-axis. (TIF) [file pone.0130328.s001.tif]

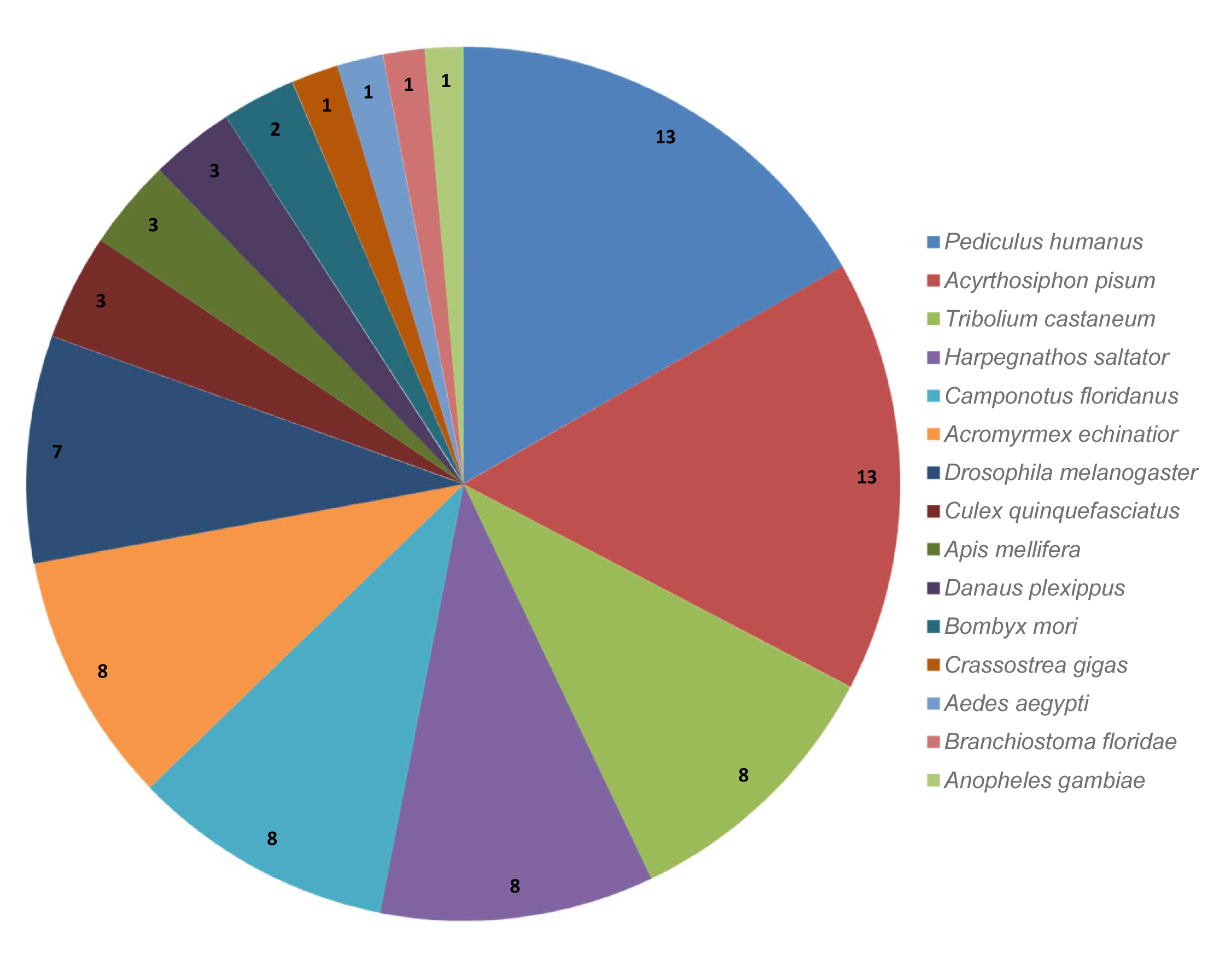

Supplement: S2 Fig — Insects comprised the top ten invertebrate species that enabled annotation of the ACP transcriptome. (TIF) [file pone.0130328.s002.tif]
